# In Search for the Genetic Basis of Quality of Life in Healthy Swedish Women—A GWAS Study Using the iCOGS Custom Genotyping Array

**DOI:** 10.1371/journal.pone.0140563

**Published:** 2015-10-15

**Authors:** Dounya Schoormans, Hatef Darabi, Jingmei Li, Yvonne Brandberg, Mikael Eriksson, Koos H. Zwinderman, Mirjam A. G. Sprangers, Per Hall

**Affiliations:** 1 Department of Medical Epidemiology and Biostatistics, Karolinska Institutet, Stockholm, Sweden; 2 Department of Medical Psychology, Academic Medical Center, Amsterdam, The Netherlands; 3 Department of Medical and Clinical Psychology, Tilburg University, Tilburg, The Netherlands; 4 Human Genetics, Genome Institute of Singapore, Singapore, Singapore; 5 Department of Oncology-Pathology, Karolinska Institutet, Stockholm, Sweden; 6 Department of Clinical Epidemiology and Biostatistics, Academic Medical Center, Amsterdam, The Netherlands; Kunming Institute of Zoology, Chinese Academy of Sciences, CHINA

## Abstract

**Background:**

Quality of life (QoL) is increasingly measured in both research and clinical practice. QoL-assessments are built on a long, empirically-based, and stringent approach. There is ample evidence that QoL is, in part, heritable. We therefore performed a GWAS relating genetic variation to QoL in healthy females.

**Methods:**

In 5,142 healthy females, background characteristics (e.g. demographic, clinical, lifestyle and psychological factors) and QoL by means of the EORTC QLQ-C30 were measured. Moreover, women were genotyped using a custom array including ~210,000 single nucleotide polymorphisms (SNPs). Initially, SNPs were related to each QoL-domain, by means of partially adjusted (controlling for age and population stratification) and fully adjusted (controlling for age, population stratification, and background characteristics) regression analyses. Additionally, gene-based analyses were performed relating the combined effect of SNPs within each gene to QoL using the statistical software package VEGAS.

**Results:**

None of the associations between QoL and genetic variation (i.e. individual SNPs and genes) reached the bonferroni corrected significance level.

**Conclusion:**

Reasons for a lack of association between genetic markers and QoL could be low variation in QoL-scores; selecting genetic markers not tagging QoL; or that the genetic effect that impacts one’s QoL is mediated through biological pathways rather than the effect of single SNPs or genes. Therefore, we opt for a pathway-based or system biology approach as a complementary and powerful approach to analyze the combined effect of genes and their biological implications in future studies focusing on QoL-issues.

## Introduction

Patient-reported outcomes are measurements based on the report that comes directly from the person, without the amendment or interpretation of others.[1] The most popular and often used patient-reported outcome is quality of life (QoL). Although many definitions of QoL exist, there is consensus that it entails at least physical, psychological, and social functioning.[2] QoL is increasingly measured in both research and clinical practice. QoL-assessments are built on a long, empirically based stringent approach. In short, QoL-measures are as reliable as other (clinical) outcomes;[3] have strong prognostic value for mortality and poor health outcomes;[4–6] and is easily measurable over time[7].

There is ample evidence that a genetic predisposition contributes to one’s QoL.[8] Several studies describe associations between QoL and the hypothalamic-pituitary-adrenal axis, immune, neuroendocrine, and cardiovascular systems.[9] In addition, genetic determinants are well established for depression, well-being, pain, and fatigue.[10–13] Furthermore, family and twin-studies have shown that the heritability for subjective well-being, depression and anxiety ranges from thirty to as much as fifty percent.[11,14,15] Beside genetics, QoL is impacted by demographic characteristics (e.g. age, sex, and race), lifestyle factors (e.g. diet and smoking), physical health and psychological factors, such as mood states, and stress.[16–19]

An international and interdisciplinary Consortium for Genetics and Quality of Life Research (GENEQOL) was instigated in 2009[8] aiming to identify biological mechanisms, genes and genetic variants involved in QoL. The studies conducted since the start showed that QoL is related various single nucleotide polymorphisms (SNPs) in cytokine genes and the glutathione metabolic pathway in various patient groups.[20–22] In a previous study based on healthy females, we related individual SNPs and the combined effect of SNPs within 139 a priori selected genes to QoL.[23] We found only one significant relation. Cognitive functioning was associated with variations in the *GSTZ1* gene.[23] For the other genes, no significant association with QoL was found. A possible explanation for the absence of associations might have been the limited approach of only including candidate genes. Therefore, we opt for a more agnostic approach in the present study. Using the same sample of healthy females, a GWAS study was performed relating genes to QoL. More specifically, the objectives were to (1) relate individual SNPs for each gene to QoL; and (2) relate the combined effect of SNPs within each gene to QoL.

## Methods

### Study population and procedure

Data from the Karolinska Mammography Project for Risk Prediction of Breast Cancer (KARMA) was used. Data used in this manuscript is available upon request through the KARMA Research Platform which can be found at www.karmastudy.org. Women who attend mammography screening or a clinical mammography at one of four Swedish participating hospitals are invited to participate in KARMA. In Sweden, the national screening program invites all women at 18 months intervals for those 40–55 years, and for those aged 56–74 years at 24 months. At each visit women’s blood is donated and processed at the Karolinska biobank. Moreover, all women are invited to complete a comprehensive online survey. This survey addresses breast cancer related issues such as reproductive history, cancer treatment, and family history of cancer; lifestyle factors (e.g. alcohol and tobacco use); previous medical conditions other than breast cancer; medication use; and QoL. The Swedish regional ethical board at the Karolinska Institutet has approved this study which was conducted in accordance with the Declaration of Helskini.[24] Women diagnosed with breast cancer before entering KARMA were excluded from this study. All women gave written consent.

### Measurements

#### Background characteristics

*Demographic and clinical factors*: Participants reported age, educational level, the use of common over-the-counter pain killers (e.g. paracetamol and ibuprofen) and whether they were on hormone replacement therapy (yes/no) during the last year. Women reported the presence of previous or ongoing medical conditions such as high blood pressure, hyperlipidemia, heart infarct, angina, heart failure, stroke, polycystic ovary syndrome (PCOS), pre-eclampsia, depression, diabetes, bulimia, and anorexia.

*Life style factors*: Body Mass Index (BMI) was calculated based on women’s weight in kilogram divided by their squared length in meters. Current tobacco use (yes/no), that is, whether women either smoked cigarettes or used snuff (a typical Swedish tobacco in moist powder form) was self-reported.

*Psychological factors*: All participants indicated there experienced level of stress during the last five years on a four-point Likert-scale ranging from *‘never stressed’* to *‘always stressed’*. They were also asked to indicate whether they have experienced any of the following life stressors during the last five years: a close relative who died; own divorce or separation; a close friend who died; serious disease or injury; became unemployed; other very stressing event. Moreover, they reported the average number of hours of sleep per night.

#### Quality of life

Patient filled out the cancer-specific QoL-questionnaire, the European Organization for Research and Treatment of Cancer Quality of Life questionnaire Core 30 (EORTC QLQ-C30).[25] It includes global health status and the following five functional scales (physical, role, emotional, cognitive, and social functioning), three symptom scales (fatigue, nausea or vomiting, and pain), and six single items (dyspnea, insomnia, appetite loss, constipation, diarrhea, and financial difficulties). These scales are linearly transformed to a scale ranging from 0 to 100. High scores indicate a high level of QoL, functioning or symptomatology. To reduce the number of tests, we included the most important QoL-domains for healthy females; the global health status and the five functional scales.[26,27] The EORTC QLQ-C30 has been validated yielding good psychometric properties.[25]

#### Genotyping

A portion of the KARMA participants was genotyped using the iCOGS chip as being a part of the iCOGS project (www.nature.com/iCOGS). This chip was specifically designed to evaluate genetic variants associated with the risk of breast, ovarian and prostate cancer.[28,29] It consists of 174,574 SNPs, selected in samples from large case-control studies in disease-based consortia.

A genome-wide imputation of SNPs using the 1000 Genomes Project (1KGP) March 2012 release (updated April 19, 2012) was performed.[30] For the KARMA dataset, the genotypes of 4,310,392 SNPs were successfully called and passed quality control filter (INFO score from IMPUTE > = 0.8 and minor allele frequency > = 0.01).

### Statistical analyses

#### Relating individual SNPs to quality of life

SNPs were related to each QoL-domain, by means of regression analyses. We chose to perform principal component analysis to correct for population stratification as uniform adjustment applied by genomic control may be insufficient at markers having unusually strong differentiation across ancestral populations and may be superfluous at markers devoid of such differentiation, leading to a loss in power.[31] The lambda GC values for each analysis after adjusting for age and an appropriate number of principle components ranged from 1.00–1.03, which suggested no remaining evidence of population stratification. PCA plots were visually inspected for outliers in terms of ancestry from CEU (northern and western Europe) clusters. Inspection of the Scree plot showed five principal components which were included in subsequent analyses. Background characteristics (Table 1), significantly related (p<0.10) to QoL based on previous analyses in this sample (S1 Table: *The association between background characteristics and quality of life using Wald Chi Square test-statistic*), were included as covariables; see Schoormans et al.[23] for a detailed description. Both partially adjusted (controlling for age and principal components) and fully adjusted regression analyses (controlling for age, principal components and covariables) were run. The statistical program PLINK was used to run analyses.[32] Bonferroni correction for multiple testing was used to define the target p-value of 2.86E-07 (0.05/174,574 SNPs). The distribution of scores was non-normal for four of the six QoL-domains. Scores on the cognitive functioning scale were transformed using square root transformation [√(101-raw score)]. On the remaining three domains (i.e. physical functioning; role functioning; and social functioning) a large percentage of women (range from 66.6% to 74.5%) reported the maximum score. These domains were therefore dichotomized; maximum value versus the remaining answers.

**Table 1:** Background characteristics and quality of life scores (n = 5142).

	N (%)
**Background characteristics**	
*Demographic factors*	
Age in mean years (range)^a^	54.3 (22–88)
Educational level^b^	
Nine year school	497 (9.7)
Gymnasium	1688 (32.9)
University	2525 (49.2)
Other	419 (8.2)
*Clinical factors*	
Being on hormone replacement therapy	1709 (33.2)
Using painkillers	4931 (95.9)
Number of medical conditions^c^	
None	2746 (53.4)
One	1509 (29.3)
Two	618 (12.0)
Three	201 (3.9)
Four or more	68 (1.3)
*Lifestyle factors*	
Body mass index (BMI) as mean score (range)^d^	25.22 (17–52)
Using tobacco	684 (13.3)
*Psychological factors*	
Stress in the last five years^e^	
Never stressed	275 (5.4)
Seldom stressed	1849 (36.4)
Often stressed	2379 (46.9)
Always stressed	571 (11.3)
Number of life stressors	
0	1728 (33.6)
1	2027 (39.4)
2	955 (18.6)
3	343 (6.7)
≥ 4	89 (1.7)
Hours of sleep^f^	
5 hours or less	207 (4.4)
6 hours	1103 (23.2)
7 hours	2170 (45.7)
8 hours or more	1269 (26.7)
**Quality of life**	
Global health/ quality of life, mean (SD)	75.8 (22.2)
*Functional scales*	
Physical functioning (highest QoL)	3427 (66.6)
Role functioning (highest QoL)	3825 (74.5)
Emotional functioning, mean (SD)	76.1 (22.8)
Cognitive functioning, mean (SD)^g^	87.8 (19.2)
Social functioning (highest QoL)	3826 (74.5)

Note

^a^ = information is missing for 1 participant

^b^ = information is missing for 14 participants

^c^ = High blood pressure and depression are the most common conditions

^d^ = for 17 participants information was unavailable

^e^ = for 68 participants no information was available

^f^ = information is missing for 393 participants. For global health/quality of life and the functional scales a higher score indicates a better quality of life. For the continuous variables (i.e. global health/quality of life; emotional functioning; and cognitive functioning) mean scores (SD) are presented. For the dichotomized scales (i.e. physical functioning; role functioning; and social functioning) frequencies and percentages for the category with the highest quality of life is provided. Please note that for the QoL-scales 6, 3, 10, 1, 0, 6 participants respectively information was missing.

^g^ = cognitive functioning was transformed by using square root transformation [√(101-raw score)], ranging from 1–10 with low scores having a better cognitive functioning. The transformed mean score and standard deviation for cognitive functioning is 2.9 (2.4).

Power calculation was done using the pwr.f2.test function in pwr package in R. The degrees of freedom for the partially and fully adjusted setting were 7 (five PCA’s, age and the outcome variable) and 13 (adding additional covariates) in the numerator and 5127 and 4681 in the denominator respectively. The significance level was set to the Bonferroni corrected level and the effect size was estimated as the fraction between the explained variability reflected by the coefficient of determination and the unexplained variability as one minus coefficient of determination from the respective model. Estimated power was 99% and 100% in the partial and fully adjusted analysis respectively, for an effect size that’s represented with assessing association of top variant with the global health/QoL-scale.

#### Relating the combined effect of SNPs within genes

Gene-based analyses were performed relating the combined effect of the 174,574 SNPs within each gene to QoL. In total, 16,512 genes were related to each of the QoL-domains separately, using the Versatile Gene-based Association Study (VEGAS) software.[33] This software package applies a test by using simulations from the multivariate normal distribution by incorporating information on a set of SNPs within a gene while accounting for linkage disequilibrium (LD) between SNPs. VEGAS uses HapMap populations to estimate patterns of LD.[33] The number of simulations for each gene is determined adaptively. In the first step, 1000 simulations are run. If the empirical value is <0.01, the number of simulations are increased to 10,000. If the empirical p-value is <0.001, another 1,000,000 simulations will be run. The simulations will continue until an empirical p-value of 0 is reached.

## Results

### Background characteristics and quality of life

In total, for 5,142 healthy women QoL and genotype data was available, hence they were included in this study. Information on background characteristics and QoL scores are described in our previous study[23], see Table 1. Mean age was 54 years. The majority of women had finished gymnasium (32.9%) or university (49.2%). A representative number of women was on hormone replacement therapy (33%).[34] Almost half of the women (46.6%) reported to be diagnosed with at least one medical condition. With respect to QoL, KARMA women were representative for the Swedish female population[23]: global health (mean = 75.8), emotional (mean = 76.1) and cognitive functioning (mean = 87.8). For the dichotomized scales, the majority of women report the highest score; 66.6% for physical functioning, and 74.5% for role and social functioning scales.

### Relating individual SNPs to quality of life

Table 2 shows the results of the association study relating individual SNPs to QoL. In both the partially adjusted (displayed on the left) and the fully adjusted models (displayed on the right), none of the SNPs were significantly related to QoL. The strongest association in our data was observed between physical functioning and rs17814073 (partially adjusted p = 2.06E-06, fully adjusted p = 1.15E-04).

**Table 2:** Relation between quality of life and single nucleotide polymorphisms (n = 174,598).

						Partially adjusted		Fully adjusted		
	top SNP	Chr	Position	Minor/ Major	MAF	Beta(SE)	p	Beta(SE)	p	GENE
**Quality of life**										
*Global health/ QoL*	rs813299	15	31438303	A/C	0.31	1.96(0.44)	7.45E-06	1.62(0.40)	5.69E-05	FAM7A1:TRPM1
*Functional scales*										
Physical functioning	rs17814073	12	70570216	A/C	0.08	0.41(0.09)	2.06E-06	0.35(0.09)	1.51E-04	CNOT2^Ɨ^
Role functioning	rs2028913	2	77252200	C/G	0.44	-0.19(0.05)	2.25E-05	-0.20(0.05)	5.21E-05	LRRTM4
Emotional functioning	rs811722	14	45181337	T/C	0.47	1.91(0.44)	1.22E-05	0.16(0.39)	2.68E-03	C14orf28^Ɨ^
Cognitive functioning^a^	rs555513	13	101927864	A/G	0.32	0.21(0.05)	3.06E-05	0.19(0.05)	6.99E-05	NALCN
Social functioning	rs17599095	4	163007597	T/C	0.10	-0.33(0.07)	7.06E-06	-0.33(0.06)	4.33E-05	FSTL5

Note: In total 174,574 SNPs were available on the imputed iCOGS chip. Bonferroni p-value = 0.05/174,598 = 2.86E-07. For the continuous variables (i.e. global health/quality of life; emotional functioning; and cognitive functioning) linear regressions were performed. For the dichotomized variables (i.e. physical functioning; role functioning; and social functioning) we used logistic regression analyses. Chr = chromosome; Position = position of the chromosome; Minor/major = minor and major alleles based on forward strand and minor allele frequencies in Europeans; MAF = minor allele frequency over all European controls in iCOGS; Beta = beta value for the minor allele relative to the major allele; SE = standard error; p = p-value.

^a^ = cognitive functioning was transformed by using square root transformation [√(101-raw score)] ranging from 1–10, with low scores having a better cognitive functioning, therefore the direction of the relation is reversed.

^Ɨ^ = the gene to which this SNP belongs is unknown, the closest gene is reported.

### In-silico functional annotation of the top SNPs and their genes

The in-silico functional annotation of the top SNPs reported in Table 2 was identified. To do so, we utilized the online databases HaploReg V2 and V3 *[* www.broadinstitute.org/mammals/haploreg] to mine ENCODE and RoadMap data; choosing to display results for variants that are in complete LD (D’ = r^2^ = 1) with the query SNPs in 1000G Phase 1 European population. The intronic variant rs813299 is located in 44kb 5' of TRPM1 gene on chromosome 15 and is predicted to alter affinity of two transcription factor binding sites; with no other variants in complete LD with it. Variant rs17814073 is located on chromosome 12 at 67kb 5' of CNOT2 gene and is in complete LD with 15 other variants spanning a ~ 51.5 Kb region of which five were in enhancer regions of several cell lines and DNAse hypersensitive sites; collectively they alter affinity of numerous transcription factor binding sites. Intronic variant rs2028913 in LRRTM4 gene was in complete LD with two other variants on chromosome 2 for which one of the variants, rs765572, altered affinity of ten binding sites and was located in histone enhancer region in adult liver cell lines. Variant rs811722 located at 185kb 5' of C14orf28 on chromosome 14 were predicted to lie in conserved region which is a DNAse hypersensitive site in two different cell lines; so was the intronic variant rs55513 of NALCN on chromosome 13. Introninc variant of FSTL5 (rs17599095) was in complete LD with four other variants spanning a region of ~ 11Kb on chromosome 4 for which they altered affinity of different set of transcription binding sites. In addition, the specific function for each gene reported in Table 2 was identified. The TRPM1 (15q13.3) gene is the founding member of the melastatin-related transient receptor (TRPM) channel family; contains 29 exons; and is a protein-coding gene that may play a role in metastasis suppression [UniProtKB(http://www.uniprot.org): Q7Z4N2]. Diseases associated with TRPM1 include melanoma metastasis, and congenital stationary night blindness, type 1c [http://www.genecards.org]. The CNOT2 (12q15) gene is a protein-coding gene which is the subunit 2 of CCR4-NOT Transcription Complex which is linked to various cellular processes including bulk mRNA degradation, miRNA-mediated repression, translational repression during translational initiation and general transcription regulation [UniProtKB(http://www.uniprot.org): Q9NZN8]. The LRRTM4 (2p12) gene is a protein-coding gene that may play a role in the development and maintenance of the vertebrate nervous system [UniProtKB(http://www.uniprot.org): Q86VH4]. The Chromosome 14 Open Reading Frame 28 (C14orf28) at 14q21.2 is a protein-coding gene that is uncharacterized [UniProtKB(http://www.uniprot.org): Q4W4Y0]. The FSTL5 at 4q32.3 is a protein-coding gene which is an important paralog to the FSTLB1 (3q13.33) gene that may modulate the action of some growth factors on cell proliferation and differentiation [UniProtKB(http://www.uniprot.org): Q12841/Q12841].

### Relating the combined effect of SNPs within genes

Results of the partially adjusted gene-based tests are provided in Table 3. All associations were non-significant (bonferroni-corrected p-value = 2.86E-07), p-values ranged from 3.34E-04 for the association between emotional functioning and *TACSTD2* to 1.01E-05 for the relation between physical functioning and the *CNOT2* gene.

**Table 3:** Gene-based test for the single nucleotide polymorphisms.

Quality of life	Chr	Gene	nSNPs	Start pos	End pos	p-value
*Global health/ QoL*	12	WNT5B	26	1596482	1626639	1.94E-04
*Functional scales*						
Physical functioning	12	CNOT2	13	68923043	69035040	1.10E-05
Role functioning	6	WRNIP1	13	2710664	2730978	1.35E-04
Emotional functioning	1	TACSTD2	10	58813682	58815754	3.34E-04
Cognitive functioning	18	ST8SIA5	10	42513078	42591037	2.65E-04
Social functioning	4	FSTL5	24	162524498	163304636	9.50E-05

Note: Bonferroni corrected p-value of 3.03E-06 (0.05/16512 genes). Chr = Chromosome; nSNPs = number of SNPs; test stat = test statistic.

## Discussion

No statistically significant associations between genetic variations (individual SNPs and combined effects of SNPs within genes) and QoL were found in the present study. There are various reasonable grounds for the absence of associations in our sample. First, it is plausible that the null findings result–at least in part–from the limitations inherent to our study. Due to our healthy female sample there was little variation in QoL scores. In addition, the dispersion over the entire genome may be skewed, since genotyping was performed by using the imputed iCOGS chip, which was originally built to identify the genetic risk for breast, ovarian and prostate cancer. Furthermore, other factors that were not measured could also impact variation in QoL, either directly or via genes. A detailed description of these plausible relations are presented in the adapted model developed by Wilson and Cleary provides.[9] In particular, associations between QoL and genes may depend on the environmental context (gene*environment interactions) which differs across populations.[35] Finally, it is important to note, that this GWAS was performed in a single study. To the best of our knowledge no independent datasets are available in which both genetic and QoL-information is available. Hence, validation was not optional. Given the continuously developing field, we believe that the number of studies will increase in the near future enabling validation of our findings. We would also like to address strengths of this study. This is the first GWAS which relates QoL to genes using a large sample of healthy females. As we control for background characteristics including self-reported chronic morbidities, we minimize the impact of illnesses. Moreover, although factors known to impact QoL (e.g. mood) are not measured, the large sample ensures us that effects of these factors on QoL are cancelled out. Furthermore, the included sample of healthy women is representative for the general Swedish population in terms of QoL, increasing generalizability of the results.

Whereas we did not find evidence for a genetic predisposition for QoL, it is likely that some variation in QoL is the result of alterations in various genes that impact one or more biological pathways, rather than the effect of single SNPs or genes. In related fields, such as psychiatric genetics, there is an abundance of studies demonstrating small effect sizes for individual genes,[10] which are difficult to replicate[36]. These individual SNP association analyses evaluate the significance of individual SNPs and offer therefore limited understanding of the complex phenotype. For future GWAS we therefore propose a system biology approach, considering not only SNPs and genes, but also pathways. A pathway-based or system biology approach is a complementary and powerful approach to analyze the combined effect of genes and their biological implications.[37,38] This strategy could also be useful in unraveling pathways that include information on gene functions and molecular mechanisms that are involved in QoL. With this new approach three important steps need to be taken. First, pathways that could be related to QoL should be identified. Second, genes for each pathway need to be determined. Genes with similar functions interact with each other more closely in the protein-protein interaction (PPI) networks than functionally unrelated genes.[39] Likewise, phenotypes are often caused by interacting functionally related genes.[37] Third, the actual statistical testing needs to be based on new methods were SNP effects are combined to represent gene effects in which subsequently they are combined into pathways. One technique is to combine p-values of all SNPs within a gene into one single p-value, which is similar to our approach using VEGAS, and then combine the p-values of all genes within one pathway to test the overall association between a pathway and QoL.[40,41] Although inspiring and hopeful, there are obvious limitations to this approach. Currently, several possible biological pathways have been suggested to be involved in QoL.[42] The selection of these pathways was formulated based on known relations between genes and QoL-domains, such as pain and fatigue. The identified biological pathways can therefore be seen as a candidate genetic-pathway approach. On a GWAS level however, the literature on epigenetic QoL-research is still in its infancy. Hence, this research field is currently too immature to formulate meaningful pathways. Moreover, pathway analysis relies on the accuracy and completeness of pathway annotation databases, such as KEGG, BioCarta, and the human interactome. That said, pathway-based analyses remain essential to gain in-depth knowledge of molecular mechanisms of QoL. In conclusion, in the current study we did not find evidence for a relation between genes and QoL. Further research is needed, as genetic markers of QoL will be valuable in clinical settings. Identification of persons susceptible to impairments in their QoL can be done by means of indicator genes and pathways. This is especially insightful when assisting persons under duress, for example when patients are being treated for a life threatening illness. When choosing treatments, clinicians may be guided by this information, opting for minimally invasive treatments when it comes to QoL and providing additional support to those patients who need it. Furthermore, by understanding the biological mechanisms underlying poor QoL, improvements may be feasible by interventions at a molecular level.

## Supporting Information

S1 Table The association between background characteristics and quality of life using Wald Chi Square test-statistic. (DOCX)
