# Supplementary material for: In Search for the Genetic Basis of Quality of Life in Healthy Swedish Women—A GWAS Study Using the iCOGS Custom Genotyping Array
Source: PLoS One. 2015 Oct 15;10(10):e0140563. doi: 10.1371/journal.pone.0140563 (PMC4607154; doi:10.1371/journal.pone.0140563)
Supplement: S1 Table — (DOCX) [file pone.0140563.s001.docx]

*Table S1: The association between background characteristics and quality of life using Wald Chi Square test-statistic*

|  | **BACKGROUND CHARACTERISTICS** | | | | | | | | | |
| --- | --- | --- | --- | --- | --- | --- | --- | --- | --- | --- |
|  | **Demographic factors** | | **Clinical factors** | | | **Lifestyle factors** | | **Psychological factors** | | |
| **QUALITY OF LIFE** | *Age* | *Educational level* | *Using painkillers* | *Number of medical conditions* | *Hormone replacement* | *BMI* | *Using tobacco* | *Stress in the last 5 years* | *Number of life stressors* | *Hours of sleep* |
| Global health/QoL | 8.97* (+) | 5.18 | 9.82* (+) | 135.18* (-) | 0.20 | 0.10 | 22.79* (-) | 410.13* (-) | 109.77*(-) | 68.06* (+) |
| **Functioning scales** |  |  |  |  |  |  |  |  |  |  |
| Physical functioning | 85.14* (-) | 1.41 | 0.58 | 83.68* (-) | 8.75* (-) | 0.81 | 12.15* (-) | 42.43* (-) | 51.18* (-) | 14.20* (+) |
| Role functioning | 0.19 | 3.40 | 2.23 | 50.70* (-) | 2.39 | 0.08 | 0.15 | 126.31* (-) | 70.75* (-) | 18.51* (+) |
| Emotional functioning | 39.64* (+) | 2.47 | 4.29* (+) | 44.73* (-) | 0.19 | 1.10 | 4.55* (-) | 902.04* (-) | 244.73* (-) | 106.80* (+) |
| Cognitive functioning^a^ | 2.16 | 0.62 | 6.62* (-) | 101.19* (+) | 3.55* (+) | 0.46 | 0.31 | 502.70* (+) | 96.86* (+) | 83.37* (-) |
| Social functioning | 10.07* (+) | 5.99 | 5.48* (+) | 118.48* (-) | 2.88* (-) | 0.08 | 1.33 | 163.28* (-) | 85.95* (-) | 19.12* (+) |
| **Symptom scales/items** |  |  |  |  |  |  |  |  |  |  |
| Fatigue | 21.49* (-) | 6.70*(+) | 5.30* (-) | 138.78* (+) | 1.66 | <0.01 | 7.20* (+) | 375.41* (+) | 154.88* (+) | 122.52* (-) |
| Nausea and vomiting | 13.60* (-) | 0.56 | 0.82 | 19.96* (+) | 5.99* (+) | 0.59 | 5.32* (+) | 50.50* (+) | 39.66* (+) | 24.01* (-) |
| Pain | 1.32 | 0.74 | 3.13* (-) | 132.70* (+) | 9.25* (+) | 0.62 | 8.40* (+) | 93.09* (+) | 102.54* (+) | 60.31* (-) |
| Dyspnoea | 42.06* (-) | 3.75 | 0.02 | 57.17* (+) | 0.35 | 1.26 | 3.71* (+) | 15.59* (+) | 17.60* (+) | 11.10* (-) |
| Insomnia | 18.29* (+) | 1.55 | 0.64 | 47.05* (+) | 6.22* (+) | 1.15 | 3.40* (+) | 187.83* (+) | 83.64* (+) | 839.61* (-) |
| Apetite loss | 5.79* (-) | 2.06 | 1.65 | 27.27* (+) | 0.63 | 1.02 | 20.79* (+) | 50.73* (+) | 37.51* (+) | 39.42* (-) |
| Constipation | 0.51 | 6.19 | 3.38* (-) | 16.76* (+) | 5.46* (+) | 1.21 | 1.08 | 20.98* (+) | 18.42* (+) | 22.32* (-) |
| Diarrhoea | 0.66 | 1.58 | 0.15 | 43.24* (+) | 5.24* (+) | 0.37 | 8.04* (+) | 40.79* (+) | 10.08* (+) | 23.76* (-) |
| Financial difficulties | 8.83* (-) | 5.27 | 3.21* (-) | 86.01* (+) | 0.35 | 0.05 | 7.23* (+) | 42.48* (+) | 88.71* (+) | 38.85* (-) |

Note: Data is presented as Wald Chi Square test-statistic. The direction of the relation is given between brackets. All background characteristics with a p-value of 0.10 were selected as possible covariables and are marked with an *. For the continuous variables (i.e. global health/quality of life; emotional functioning; cognitive functioning; fatigue; pain; dyspnoea; and insomnia) linear regression analyses were performed. For the dichotomized variables (i.e. physical functioning; role functioning; social functioning; nausea and vomiting; appetite loss; constipation; diarrhea; financial difficulties) we used logistic regression analyses. ^a^ = cognitive functioning was transformed by using square root transformation [√(101-raw score)], ranging 1-10 with low scores having a better cognitive functioning, therefore the direction of the relation is reversed.
